# Dual Monitoring of Secretion and ATP Levels during Chondrogenesis Using Perfusion Culture-Combined Bioluminescence Monitoring System

**DOI:** 10.1155/2015/219068

**Published:** 2015-10-29

**Authors:** Hyuck Joon Kwon, Youngbae Han

**Affiliations:** ^1^Department of Physical Therapy and Rehabilitation, College of Health Science, Eulji University, Gyeonggi 13135, Republic of Korea; ^2^Department of Mechanical and Design Engineering, Hongik University, Sejong City 30016, Republic of Korea

## Abstract

Skeletal pattern formation in limb development depends on prechondrogenic condensation which prefigures the cartilage template. However, although morphogens such as TGF-*β*s and BMPs have been known to play essential roles in skeletal patterning, how the morphogens induce prechondrogenic cells to aggregate and determine patterns of cartilage elements has remained unclear. Our previous study reported that ATP oscillations are induced during chondrogenesis. This result suggests the possibility that ATP oscillations lead to the oscillatory secretion of morphogens, due to the fact that secretion process requires ATP. To examine the correlation between ATP oscillations and secretion levels of morphogens, we have developed perfusion culture-combined bioluminescence monitoring system to simultaneously monitor intracellular ATP levels and secretion levels. Using this system, we found that secretory activity oscillates in phase with ATP oscillations and that secretion levels of TGF-*β*1 and BMP2 oscillate during chondrogenesis. The oscillatory secretion of the morphogens would contribute to amplifying the fluctuation of the morphogens, underlie the spatial patterning of morphogens, and consequently lead to skeletal pattern formation.

## 1. Introduction

Skeletal development in vertebrate limb begins with chondrogenesis in which prechondrogenic cells condense and then differentiate into chondrocytes to form a variety of precisely shaped cartilage elements that are ultimately replaced by bone tissues through endochondral ossification [1, 2]. The prechondrogenic condensation is the essential stage in skeletal pattern formation during limb development [3]. The transforming growth factor-*β* (TGF-*β*) superfamily of growth factors including TGF-*β*s and bone morphogenetic proteins (BMPs) is vital for skeletal patterning and thus is called morphogen [4–6]. However, how these morphogens regulate prechondrogenic condensation and thus determine skeletal patterns spatiotemporally remains unclear. Although the previous models for the skeletal pattern formation have proposed the existence of a molecular clock that provides positional information [7, 8], there is no evidence for the molecular clock to determine positional values during skeletal development. Another approach has suggested theoretically that small spatial fluctuations of reacting and diffusing morphogens could become unstable and that amplification of these fluctuations could lead to their spatial patterns for skeletal pattern formation [9–11]. However, the spatial patterning of morphogens by reaction and diffusion has not been experimentally proved yet.

Morphogens are extracellular secreted molecules and thus their regulation can depend on secretion process. Regulated secretion has been functionally dissected into a number of stages: priming steps, physical movement of vesicles to the subplasmalemmal region of the cell, tethering and then docking at release sites on the plasma membrane, conversion to a fully releasable state, triggered membrane fusion, release of granule contents, and finally retrieval of the granule membrane [12–14]. Intracellular ATP plays a crucial role in multiple steps of secretion process such as secretory vesicle transport, the priming process for membrane fusion, and supply of phosphate group [15, 16]. These facts indicate that energy metabolism is closely associated with the secretion process. Consistent with this fact, it has been reported that metabolic oscillations occur and induce oscillatory secretion of insulin in pancreatic *β*-cells [17, 18]. Our previous work also demonstrated that metabolic oscillations generate in early step of chondrogenesis and play an essential role in prechondrogenic condensation [19]. This suggests that the metabolic oscillations lead to oscillatory secretion of the morphogens, which subsequently controls their spatiotemporal patterns, regulates prechondrogenic condensation, and consequently determines skeletal patterns.

In this study, we have examined whether secretion activity of the morphogens oscillates during ATP oscillations in chondrogenesis. Perfusion culture system has been used for monitoring temporal change of secretion levels [20, 21]. However, the previous perfusion culture system was not capable of measuring cellular metabolic state with secretion levels and thus had limitation for clarifying the correlation between metabolic state and secretory activity. Here, we have developed the bioluminescence monitoring system combined with the perfusion culture system to monitor simultaneously both intracellular ATP level and extracellular secretion level. Using this system, we found that extracellular secretory activity oscillates in phase with ATP oscillations during chondrogenesis. Furthermore, it was shown that secretion levels of BMP2 and TGF-*β*1 oscillate during chondrogenesis and that each peak of the oscillatory secretion of BMP2 and TGF-*β*1 appears at the peak of ATP oscillations. However, the oscillatory secretion of the growth factors showed one peak per two or three peaks of ATP oscillations and thus the frequency of their oscillatory secretion was lower than that of ATP oscillations. This result indicates that the secretion patterns of the growth factors depend on not only secretory activity but also other processes. The oscillatory secretion of growth factors would play a crucial role in prechondrogenic condensation and subsequent skeletal patterning.

## 2. Methods 

### 2.1. Cell Culture and Light Microscopic Observation

When ATDC5 cells maintained in Dulbecco's modified Eagle's medium-F12 (DMEM-F12) (Gibco, Tokyo, Japan) supplemented with 5% fetal bovine serum (FBS), 10*μ*g/mL human transferrin (Roche Molecular Biochemicals, Indianapolis, IN, USA), and 3 × 10^−8^ M sodium selenite (Sigma-Aldrich, St. Louis, MO, USA) reached confluency, the medium was replaced with the medium supplemented with 10 *μ*g/mL insulin (Sigma-Aldrich). Microscopic observation was performed with a microscope (Nikon Eclipse TE300, Nikon, Tokyo, Japan).

### 2.2. Construction of Reporter Genes and Transfection

A* Phrixothrix hirtus *luciferase (PxRe) gene (Toyobo, Osaka, Japan) to express ATP-dependent luciferase emitting red light and* Cypridina *luciferase (CLuc) gene (NEB, Ipswich, MA, USA) to express secreted luciferase [22] were used to monitor intracellular ATP level and secretion levels during chondrogenesis, respectively. The human actin promoter (–500/+101, donated by Toyobo) was inserted into multiple cloning sites of vectors containing a PxRe gene and a CLuc gene, respectively. Then, the promoter region and the luciferase gene were inserted into retrovirus vectors (Clontech, Mountain View, CA, USA), respectively. ATDC5 cells were transfected using retrovirus infection and then were selected by G418 or puromycin.

### 2.3. Real-Time Monitoring of Intracellular ATP Levels

After ATDC5 cells were transfected with a PxRe gene, the medium was replaced with the medium including luciferin (0.1 mM). Bioluminescence (relative light unit: RLU) was monitored using a dish-type luminescencer, Kronos, for 1 min at 1–30 min intervals under the perfusion culture or the static culture.

### 2.4. Simultaneous Monitoring of Secretion Activity and Intracellular ATP Levels

Simultaneous monitoring of CLuc secretion and PxRe activity was performed by perfusing the medium (10 *μ*g/mL insulin and 0.05 mM luciferin) from bottles to the fraction collector at a flow rate of 1.0 mL/h and collecting the fractions every 30 min during the monitoring of PxRe activity using Kronos. The accumulated P_ACTIN_-PxRe intensity for 1 min was monitored during the perfusion culture for 1 week. After that, the secretion level of CLuc in each collected fraction was measured by mixing with* Cypridina *luciferin (1 *μ*M).

### 2.5. Real-Time Monitoring of Secretion Levels of Growth Factors from ATDC5 Cells

Simultaneous monitoring of secretion of growth factors and PxRe activity was performed by perfusing the medium (10 *μ*g/mL insulin and 0.05 mM luciferin) and the fractions were collected during the monitoring of PxRe activity using luminometer (ATTO, Osaka, Japan). After that, the BMP2 or TGF-*β*1 levels in these fractions were measured by using an ELISA kit (R&D systems Inc., USA) according to the manufacturer's instructions. To examine the effect of chemical compounds on ATP oscillations, the cells were perfused with the medium supplemented with 5 mM cyanide (Sigma-Aldrich).

## 3. Results and Discussion

### 3.1. Real-Time Monitoring of Intracellular ATP Levels during Chondrogenesis under Perfusion Culture with Chondrogenic Medium

**Figure 1:**
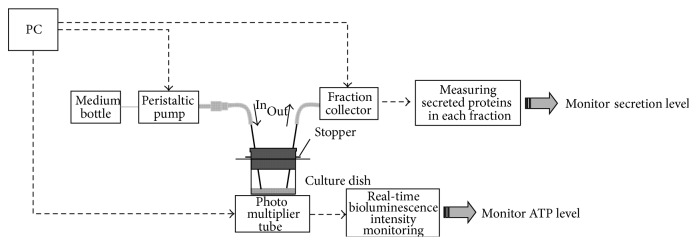
The schematic representation of perfusion culture-combined bioluminescence monitoring system.

**Figure 2:**
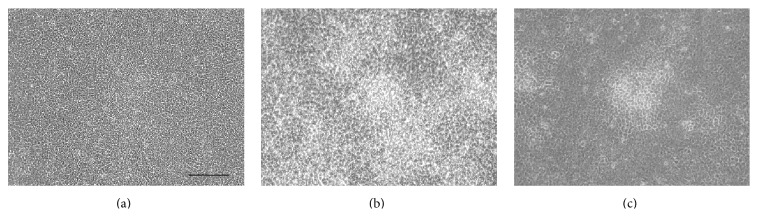
Perfusion culture increases the condensation degree compared to static culture. ATDC5 cells were observed with phase contrast microscopy after 5 days of the perfusion culture with either the maintenance medium (a) or the chondrogenic medium (b) and the static culture with the chondrogenic medium (c). Scale bars, 100 *μ*m.

**Figure 3:**
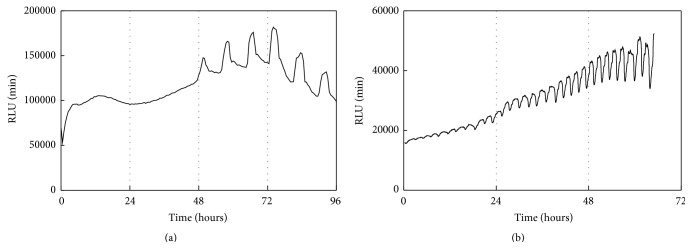
Perfusion culture induces ATP oscillations to be started earlier and to have higher frequency than static culture. While ATDC5 cells transfected with the PxRe reporter gene were cultured in the static culture with the chondrogenic medium (a) or the perfusion culture with the chondrogenic medium (b), bioluminescence intensities from the ATDC5 cells were monitored in real time.

After ATDC5 cells transfected with PxRe reporter gene grew to confluence in the maintenance medium, perfusion cultures were performed by perfusing the chondrogenic medium including D-luciferin at a flow rate of 1.0 mL/h by a peristaltic pump (Figure 1). We found that ATDC5 cells showed almost no signs of cellular damage under the perfusion culture with maintenance medium or chondrogenic medium. ATDC5 cells differentiated into cartilage nodules via condensation process under the perfusion culture with chondrogenic medium (Figure 2(b)), whereas they did not form any condensations under the perfusion culture with maintenance medium. Cellular condensations appeared much more under the perfusion culture, compared to the static culture (Figures 2(b) and 2(c)). This result can be explained by rapid progress of chondrogenesis due to continuous perfusing with fresh chondrogenic medium. In addition, PxRe activity began to oscillate 2 days after chondrogenic induction under the static culture (Figure 3(a)), whereas PxRe activity began to oscillate much earlier (within 12 hours after chondrogenic induction) under the perfusion culture (Figure 3(b)). Moreover, the frequency of PxRe oscillations under the perfusion culture was 2–4 times higher than that of PxRe oscillations under the static culture (Figures 3(a) and 3(b)). Our results showed the positive correlation between the frequency of ATP oscillations and the condensation degree and thus imply that the frequency of ATP oscillations encodes the condensation degree and subsequent skeletal pattern formation during limb development.

### 3.2. Simultaneous Monitoring of the Levels of Intracellular ATP and Secreted Luciferase during Chondrogenesis

**Figure 4:**
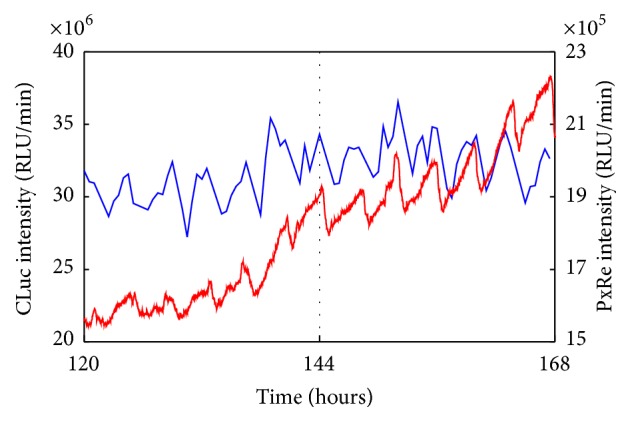
Simultaneous monitoring of PxRe intensity (red line) and secreted CLuc intensity (blue line) during perfusion with chondrogenic medium.

We transfected ATDC5 cells with both PxRe and CLuc reporter genes and then measured PxRe activities and CLuc secretion to monitor intracellular ATP levels and secretory activity simultaneously during chondrogenesis by using perfusion culture-combined bioluminescence monitoring system. We found that CLuc secretion oscillated nearly in phase with PxRe oscillations (Figure 4). This result indicates that secretory activity oscillates in phase with ATP oscillations, which can be explained by the fact that intracellular ATP is required for secretion processes [17, 18]. Therefore, this result implies that metabolic activities in cells can regulate the action of extracellular signalling molecules by controlling secretory activities and suggests the possibility that the secreted growth factors can oscillate at the secretion level during chondrogenesis.

### 3.3. Simultaneous Monitoring of the Levels of Intracellular ATP and Growth Factors during Chondrogenesis

**Figure 5:**
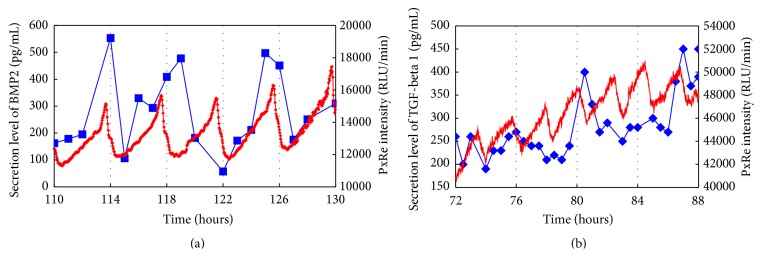
(a) Simultaneous monitoring of PxRe intensity (red line) and secreted BMP2 levels (blue line) during perfusion with chondrogenic medium. (b) Simultaneous monitoring of PxRe intensity (red line) and secreted TGF-*β*1 levels (blue line) during perfusion with chondrogenic medium.

**Figure 6:**
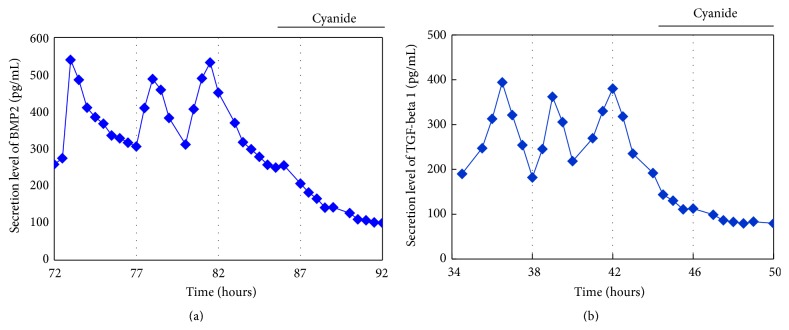
Effect of mitochondrial respiration inhibitor cyanide on the oscillatory secretion of BMP2 (a) and TGF-*β*1 (b) during perfusion with insulin-supplemented medium.

We examined whether secretion levels of BMP2 or TGF-*β*1, which are crucial morphogens for skeletal formation [4–6], oscillate with ATP oscillations during chondrogenesis. PxRe intensity was monitored during the perfusion culture with chondrogenic medium, in which we collected the fractions every 30 min and then measured the amount of secreted growth factors in each fraction by using ELISA. As a result, BMP2 showed oscillatory secretion and the oscillatory secretion of BMP2 represented the peak phase nearly at the peak phase of PxRe oscillations during chondrogenesis (Figure 5(a)). However, the BMP2 secretion occasionally did not reveal the peak phase at every peak of PxRe oscillations (Figure 5(a)). Similarly, secretion level of TGF-*β*1 also oscillated during chondrogenesis (Figure 5(b)). The oscillatory secretion of TGF-*β*1 revealed the peak phase immediately following the peak phase of PxRe oscillations but showed one peak per two or three peaks of PxRe oscillations (Figure 5(b)). To demonstrate clearly the correlation between ATP oscillations and the oscillatory secretion of the morphogens, we examined whether inhibition of ATP oscillations suppresses the oscillatory secretion of the morphogens. Our previous study showed that ATP oscillations depend on mitochondrial respiration and thus a mitochondrial respiration inhibitor cyanide inhibits ATP oscillations during chondrogenesis [19, 23]. The present result showed that cyanide suppressed the oscillatory secretion of both BMP2 and TGF-*β*1 (Figures 6(a) and 6(b)), which corroborates that ATP oscillations mediate the oscillatory secretion of these growth factors. However, the oscillatory secretion of BMP2 or TGF-*β*1 showed lower frequency than that of ATP oscillations, which was different from the result with the secreted CLuc (Figure 4). This result can be explained by the fact that not only the secretion process but also other processes such as transcriptional and translational processes regulate the secretion levels of the growth factors during chondrogenesis.

Skeletal pattern formation is determined by prechondrogenic condensation process of mesenchyme stem cells [24]. A spatially nonuniform distribution of the morphogens would be required for inducing prechondrogenic condensation in specific sites and subsequent skeletal patterning [25]. Our present study showed the oscillatory secretion of BMP2 and TGF-*β*1 during chondrogenesis. The oscillatory secretion of the morphogens can contribute to amplifying the fluctuation of the morphogens and consequently underlie the spatial patterning of morphogens. It is known that the spatial profile of the morphogens is dictated not by their receptor distribution, which is uniform, but by molecular patterns of the diffusible morphogens themselves [24]. Therefore, our data propose that the pattern formation of BMPs and TGF-*β*s which subsequently determine skeletal patterning depends on the oscillatory secretion of the morphogens themselves which is mediated by metabolic oscillations.

## 4. Conclusion

Perfusion culture-combined bioluminescence monitoring system was enabled to monitor simultaneously intracellular ATP level and secretion level of growth factors during chondrogenesis. We found that secretion levels in BMP2 and TGF-*β*1 oscillate during ATP oscillations in chondrogenesis. Our result implies that the oscillatory secretion of these morphogens would contribute to spatiotemporal control of skeletal formation. The perfusion culture-combined bioluminescence monitoring system would provide useful tool for studying the effects of metabolic regulation or genetic regulation on secretion of extracellular signalling molecules. In addition, limitations of tissue engineering approach under static culture system include insufficient nutrient and oxygen transport and waste removal which will cause decreased proliferation and differentiation and nonuniform cell distribution. The perfusion culture system may be a solution of those problems by continuously providing nutrients and oxygen and removing the waste from the cultured cells. Therefore, our perfusion culture-combined bioluminescence monitoring system would be very useful for monitoring dynamics of extracellular and intracellular molecules in a variety of tissue engineering approaches.
